# Association between Serum Level of Multiple Trace Elements and Esophageal Squamous Cell Carcinoma Risk: A Case–Control Study in China

**DOI:** 10.3390/cancers14174239

**Published:** 2022-08-31

**Authors:** Jingbing Zhang, Geng Wang, Anyan Huang, Kexin Cao, Wei Tan, Hui Geng, Xiaosheng Lin, Fulan Zhan, Kusheng Wu, Shukai Zheng, Caixia Liu

**Affiliations:** 1 Department of Preventive Medicine, Shantou University Medical College, Shantou 515041, China; 2 Department of Thoracic Surgery, Cancer Hospital of Shantou University Medical College, Shantou 515041, China; 3 Mental Health Center, Shantou University Medical College, Shantou 515065, China; 4 Health Management Center, The People’s Hospital of Jieyang, Jieyang 522000, China; 5 Department of Ultrasound, First Affiliated Hospital of Shantou University Medical College, Shantou 515041, China; 6 Department of Burns and Plastic Surgery, and Cleft Lip and Palate Treatment Center, Second Affiliated Hospital of Shantou University Medical College, Shantou 515041, China

**Keywords:** esophageal squamous cell carcinoma, trace elements, chemical carcinogenesis, mixed exposure

## Abstract

**Simple Summary:**

China accounts for half of the global new cases of esophageal cancer (EC). A disorder of individual trace elements was established as increasing risk for developing esophageal cancer, but the truth was that people are exposed to multiple metals simultaneously in the real world, and research on the relationship between mixtures of accumulative hazards and EC remains scarce. In our current research, we used BKMR, an approach designed to estimate the overall effect of a mixture, to explore the possible role of different trace elements combined in esophageal squamous cell carcinoma (ESCC) risk. In addition, some interesting results were found in our study. We hope it will provide more evidence for environment-related carcinogenesis of ESCC.

**Abstract:**

We investigated the associations between multiple serum trace element levels and risk for esophageal squamous cell carcinoma (ESCC). A total of 185 ESCC patients and 191 healthy individuals were recruited in our study. The concentration of 13 trace elements (Al, V, Cr, Mn, Co, Ni, Cu, Zn, As, Se, Sr, Cd and Pb) in serum was determined with inductively coupled plasma mass spectrometry (ICP-MS). Logistic regression and the Probit extension of Bayesian Kernel Machine Regression (BKMR) models was established to explore the associations and the cumulative and mixed effects of multiple trace elements on ESCC. Three elements (Zn, Se and Sr) displayed a negative trend with risk for ESCC, and a significant overall effect of the mixture of Al, V, Mn, Ni, Zn, Se and Sr on ESCC was found, with the effects of V, Ni and Sr being nonlinear. Bivariate exposure–response interactions among these trace elements indicated a synergistic effect between Zn and Se, and an impactful difference of V combined with Ni, Sr or Zn. Our results indicate that Ni, V, Al, Mn, Zn, Se and Sr are associated with ESCC risk, providing additional evidence of the complex effects of trace elements disorder during the etiology of EC development.

## 1. Introduction

Cancer is the leading cause of death before 70 years of age in many countries, indicating a huge disease burden on society and individuals. The International Agency for Research on Cancer (IARC) estimated about 19.3 million newly diagnosed cases and 10.0 million deaths worldwide in 2020, and esophageal cancer ranks sixth in lethal malignancy (https://gco.iarc.fr/, accessed on 31 May 2022). China accounts for half of the global new cases of esophageal cancer (EC), and esophageal squamous cell carcinoma (ESCC), the most common histopathological subtype of EC, represents about 80% of the cases [1]. 

The causes of ESCC are complicated and population-dependent. Tobacco use, alcohol consumption, hot food and hot beverage consumption, poor diet and specific food intake have been suggested as risk factors for ESCC [1,2]. Single trace element disorders, such as a deficiency in essential trace elements and exposure to toxic metals, have been established as a risk factor for developing esophageal cancer [3]. For example, a lower serum selenium status is an esophageal cancer risk factor for subjects from China [3,4], Europe [5] and Africa [6], while a higher zinc intake is associated with reduced esophageal cancer risk in Asia, but not in America and Europe [7], and Asian subjects benefit from the protective effect of dietary calcium intake against ESCC [8]. Moreover, with the development of industry, pollution of the environment and wide use of countless chemical products, trace metal exposure has become a life-long condition. A wide variety of epidemiological studies have reported associations between heavy metal exposure and cancer risk [9,10,11], but research on the effects of combined mixtures of accumulative hazards remains scarce for EC. In the previous literature on EC, most investigate single heavy metal exposure and their health outcomes [3,12]. However, the reality is that people are exposed to multiple metal pollutants simultaneously, and such exposure often has non-additive or non-linear effects on health, leading to biased or underestimated results with traditional multivariable regression analysis [13]. This is a critical concern in addressing the challenges in environmental epidemiology. Bayesian kernel machine regression (BKMR) is an approach designed to estimate the effect of overall mixtures and identify the pollutants responsible for the observed effect of mixtures or the interactions among individual pollutants [14]. In the present study, we recruited ESCC patients and their age and gender-matched healthy individuals, and quantified 13 elements in serum, including aluminum (Al), vanadium (V), chromium (Cr), manganese, cobalt (Co), nickel (Ni), copper (Cu), zinc (Zn), arsenic (As), selenium (Se), strontium (Sr), cadmium (Cd) and lead (Pb), to assess the relationships between these elements’ mixture exposure and the risk of ESCC in a southern area with high rate of EC in China, providing a reference for the etiology of EC. 

## 2. Materials and Methods

### 2.1. Study Population, Data Collection and Variable Definition

The study participants were recruited from the Cancer Hospital of Shantou University Medical College. The inclusion criteria for patients were: (1) age between 40 and 85 years old, (2) diagnosed with esophageal squamous cell carcinoma based on pathological examination, (3) have lived in southern China for more than 5 years. The exclusion criteria were: (1) preoperative radiotherapy or chemotherapy, (2) distant metastasis, (3) history of other malignancies and serious diseases of the liver or kidney. The control group involved was comprised of healthy individuals enrolled from the other two general hospitals in the same area, were matched to the cases by age and sex, and had no history of malignancy, concurrent infection, or chronic infection. This study was approved by the Ethics Committee of Shantou University Medical College (No. 20210339), and all participants signed the informed consent forms.

Demographic data of the participants were collected using a standardized questionnaire administered by two trained research staff. The information collected included sex, age, family history of EC, and smoking and drinking habits. Smoking status was considered current if they reported smoking at the initial consultation or had quit within 1 year of diagnosis, while alcohol consumption was defined as drinking on average more than three times or more than 100 mL ethanol per week [15].

### 2.2. Serum Trace Element Assay

For the ESCC group, whole blood was collected on the day before esophagectomy, whereas specimens of the control group were collected on the morning of the physical examination. All samples were centrifuged at 1000× *g* for 10 min to separate the upper layer after clotting, and the resulting serum was stored in the ultra-low temperature freezer until examination. Before pretreatment, samples were thawed and vortexed at room temperature. Then, a 50 μL aliquot was transferred to a Teflon tube and lyophilized at −130 °C for 5 h. After lyophilization, 250 μL 65% nitric acid (Merck, Darmstadt, Germany) was added and heated in an oven at 80 °C for 90 min for digestion. After cooling, the samples were diluted with ultrapure water (Milli-Q, Millipore Inc., Bedford, MA, USA) to 5 mL and analyzed by using inductively coupled plasma mass spectrometry (Agilent 7900, Agilent Technologies, Santa Clara, CA, USA). By continuously diluting a standard solution with 3% nitric acid, a 9-point calibration curve within the concentration range was created to calculate the element concentration in the samples. Thirteen elements were determined in the samples: aluminum (Al), vanadium (V), chromium (Cr), manganese (Mn), cobalt (Co), nickel (Ni), copper (Cu), zinc (Zn), arsenic (As), selenium (Se), strontium (Sr), cadmium (Cd) and lead (Pb). During pretreatment and analysis, the standard reference material for serum (Seronorm Trace Elements Serum L-2 RUO, SERO Inc., Oslo, Norway) was used for accuracy and quality control.

### 2.3. Statistical Analysis

PASS 11 (NCSS, LLC. Kaysville, UT, USA) was used to calculate the sample size based on a significance level (alpha) of 0.05 using a two-sided two-sample *t*-test. With a sample size of 184 subjects in each group, the power of the study would be more than 90% for detecting the concentration difference in serums Se and Zn, two elements previously verified to be at lower levels in EC [16].

The chi-square test was used to compare the categorical variables for the demographic characteristics between the case and control groups, and the differences in the serum trace element concentrations between the two groups were compared using the Wilcoxon rank sum test. Additionally, Spearman’s rank correlation analysis was performed to explore the correlation among the elements. Associations between serum trace element levels and the risk of ESCC were performed by binary logistic regression, while the serum trace element concentrations were converted to quartiles with the lowest quartile as the reference group. In addition, *p*-trend values were calculated with the median concentration of each quartile group using binary logistic regression analysis.

In BKMR analysis, the concentrations of trace elements in the serum were converted to the natural logarithm. The trace elements, which had statistical significance in the multiple trace elements model, were included in the Probit extension of Bayesian kernel machine regression (BKMR-P) to explore the cumulative, individual effects and interactions. In addition, we also visualized the single exposure response function and binary exposure response function, and calculated the posterior inclusion probability (PIP) of the trace elements. The PIP is a measure of the variable’s contribution to ESCC risk, with higher values (close to 1) indicating higher contributions, and lower values (close to 0) indicating lower contributions. The threshold for PIP was set as 0.5 in the current study. The overall effect was calculated by comparing the ESCC risk (the probability of developing EC) when all of the predictors were set at a particular quantile (every tenth percentage, for example) as compared to when all of the predictors were at their 50th percentile. The individual effect was evaluated by comparing the risk when a single element was set at the 75th percentile level as compared to the 25th percentile level, when all the other elements were fixed at their 25th, 50th and 75th percentile. The interaction effect was evaluated by comparing the risk when all the other elements were fixed to their 75th percentile to when all the other elements were fixed to their 25th percentile. 

Software SPSS 26.0 (IBM Corp., New York, NY, USA) and R 4.1.1 (R Core Team, Vienna, Austria) were used for statistical analysis. A two-sided *p* < 0.05 was considered as indicating statistical significance.

## 3. Results

### 3.1. General Characteristics of the Study Population

The demographic characteristics of the study participants are shown in Table 1 and Table S1. A total of 191 ESCC patients and 185 healthy individuals were enrolled in the study. The average age was 62.23 ± 8.86 years for the cases and 62.65 ± 7.36 years for the controls. In the case group, 137 participants (71.9%) were males and were comparably distributed with that of the control group (71.7%). There were also no statistical differences in the composition of age, sex and drinking status between the cases and controls (*p* > 0.05). In terms of smoking status, the percentage of smokers in the case group (65.97%) was higher than that in control group (38.25%) (*p* < 0.001). Moreover, there were also significantly more individuals with a family history of EC in the case group (*p* < 0.05).

### 3.2. Serum Trace Element Concentrations

**Figure 1:**
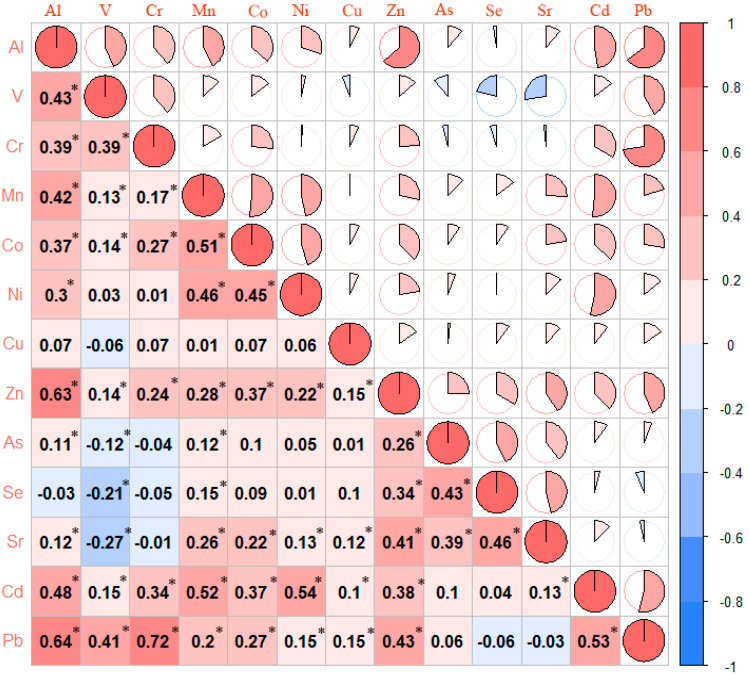
Spearman’s correlation heatmap matrix of ln-transformed serum trace element concentrations among the study participants. Abbreviations: Al, Aluminum; V, Vanadium; Cr, Chromium; Mn, Manganese; Co, Cobalt; Ni, Nickel; Cu, Copper; Zn, Zinc; As, Arsenic; Se, Selenium; Sr, Strontium; Cd, Cadmium; Pb, Lead. * *p* < 0.05.

Among all the trace elements, serum concentrations of Al, Mn, Co, Zn, As, Se, Sr, Cd and V showed significant differences between the ESCC cases and controls (*p* < 0.05) (Table 2). The levels of Al, Mn, Co, Zn, As, Se, Sr and Cd in the cases were lower than those in the control group (*p* < 0.05), while serum V concentrations of the case group were higher than that in the control group (*p* < 0.05). According to Spearman’s rank correlation analysis, we found the trace elements correlated with each other, with correlation coefficients ranging between 0.11 to 0.72 (Figure 1). Therefore, we conducted a collinearity diagnostic on the concentrations of all the trace elements (Table S2) and found no evidence of collinearity (all VIF < 4).

### 3.3. Associations of Trace Element Concentrations with Risk for ESCC

Then, we used logistic regression to analyze the association of each element’s concentration with ESCC. As presented in Table S3, after adjusting for covariates (smoking status, drinking and family history of EC), a positive relationship between the V and Ni serum concentrations and ESCC risk was found, as well as a negative relationship between the levels of Al, Mn, Co, Zn, As, Se, Sr and Cd and the probability of ESCC. Among them, Zn and Se accounted for the lowest OR (0.04 to 0.19) for ESCC with a statistically significant *p*-value for the trend.

Then, these 10 trace elements with significant differences were entered into the multi-element logistic regression model for further analysis (Table 3). After adjusting for the confounding factors, compared to the concentrations in the lowest quartile group, serum Se (OR_Q2_ = 0.18, 95% CI: 0.05–0.71; OR_Q3_ = 0.19, 95% CI: 0.05–0.74; OR_Q4_ = 0.06, 95% CI: 0.01–0.27, with *p* for trend < 0.001) significantly decreased the risk of ESCC. Inversely, there was a positive association between serum Ni concentrations and risk of ESCC for Ni concentrations in the second and third quartile groups (OR_Q2_ = 5.95, 95% CI: 1.74–20.33; OR_Q3_ = 4.60, 95% CI: 1.31–16.22, *p* trend = 0.008). Moreover, serum Al in the third quartile showed an 85% reduced OR in cases (95% CI: 0.03–0.75), though no statistically significant trend was found. On the contrary, the serum V increased the risk for EC when the concentration of V was in the third quartile (OR_Q3_ = 7.16, 95% CI: 1.70–30.15). Zn (OR_Q3_ = 0.04, 95% CI: 0.01–0.21; OR_Q4_ = 0.12, 95% CI: 0.02–0.86) and Sr (OR_Q3_ = 0.11, 95% CI: 0.03–0.40; OR_Q4_ = 0.001, 95% CI: 0.00–0.01) both presented a decreased risk for ESCC when their concentrations were in the third and the highest quartile, with a *p* value of less than 0.001. Compared to the unadjusted model, after adjusting for the covariates, better protective effects of Se and different impacts of the Zn level, a change risk of V and Ni was found for ESCC.

### 3.4. Bayesian Kernel Machine Regression (BKMR) Analyses

**Figure 2:**
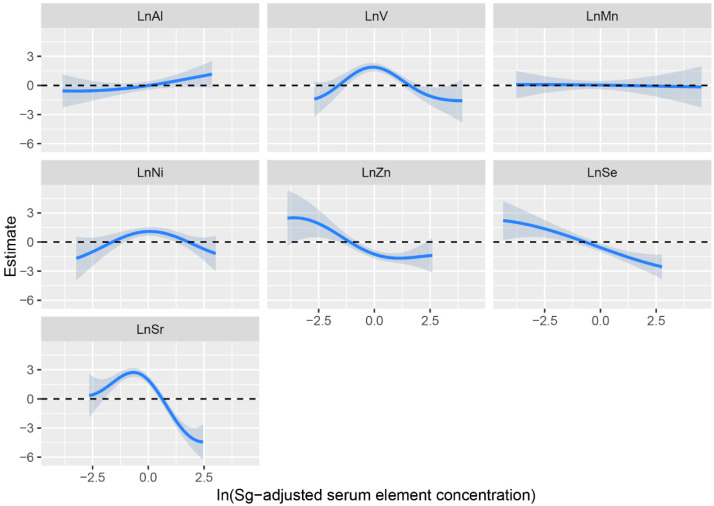
Univariate exposure–response relationship of serum Al, V, Mn, Ni, Zn, Se and Sr concentrations. The model was adjusted for smoking, drinking and family history of esophageal cancer, and other elements were fixed at their median.

Based on the results of the multivariate logistic regression model, we further selected seven trace elements (Al, V, Mn, Ni, Zn, Se, Sr) for BKMR analysis. Figure S1 shows that Al, V, Mn, Ni, Zn, Se and Sr were of high importance in the model, especially for V, Ni, Zn, Se and Sr (all PIP = 1.00). A linear relationship between ESCC risk and individual exposures to Al, V, Ni, Zn, Se and Sr was found when other elements were fixed at the median (Figure 2). A negative trend was found for ESCC risk with serum Se concentrations, which is consistent with the results of multi-element logistic regression, whereas the dose–response curve for Zn tended to be linear but was smooth slightly at lower or elevated concentrations. Notably, the dose–response relationship between ESCC risk and serum V, Ni and Sr concentration was an inverted U-shape, whereas serum Al concentration showed a positive trend with ESCC. A dose–response relationship between serum Mn concentration and EC risk was barely observed.

**Figure 3:**
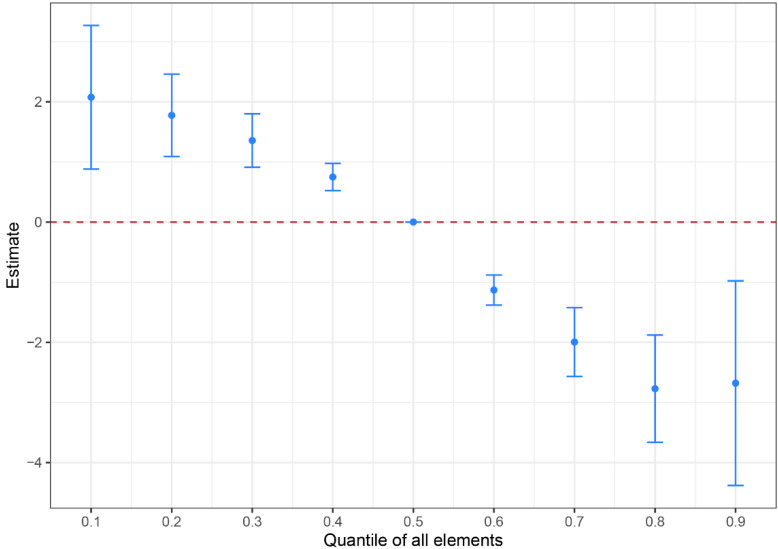
Overall effect of the seven elements on the risk of EC (Al, V, Mn, Ni, Zn, Se, Sr). The model was adjusted for smoking, drinking and family history of esophageal cancer.

A statistically significant overall effect of the seven trace elements on the risk for ESCC is shown in Figure 3. When the overall mixture was at or lower than the 40th percentile, exposure to the mixture showed increased risk for ESCC as compared with their concentrations at the 50th percentile, and these elements were associated with less risk when at or above the 60th percentile compared with their concentrations at the 50th percentile.

**Figure 4:**
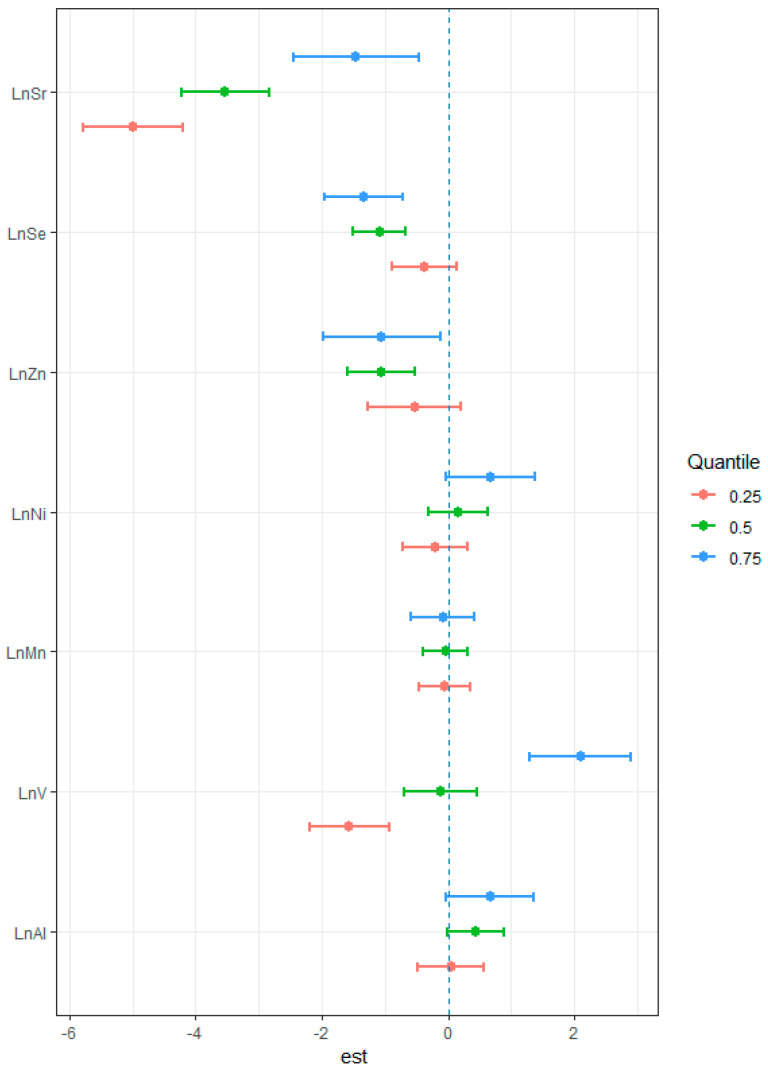
The individual effect of the seven elements on the risk of ESCC. Estimated values were calculated using the Bayesian kernel machine regression (BKMR) model by comparing the risk of ESCC when the other trace elements are fixed at their 25th (red), 50th (green) or 75th (blue) percentile. The model was adjusted for smoking, drinking and family history of EC.

The individual effects of the seven trace elements on the risk for EC are shown in Figure 4. A strong negative association between serum Sr concentrations and risk for EC was seen when the concentrations of the other trace elements were at their 25th, 50th and 75th percentiles. However, with the increase in the other trace element concentrations, this negative association was reduced. Moreover, Se and Zn had protective effects against ESCC, and the effects were statistically significant when these two elements were at their 50th and 75th percentiles. Interestingly, serum V concentration was positively associated with the risk for ESCC when the other elements were at the 75th percentiles, whereas a negative relationship was found when the other trace element concentrations were at their 25th percentiles. The results above may suggest the possibility of interaction between Sr, V and other trace elements.

**Figure 5:**
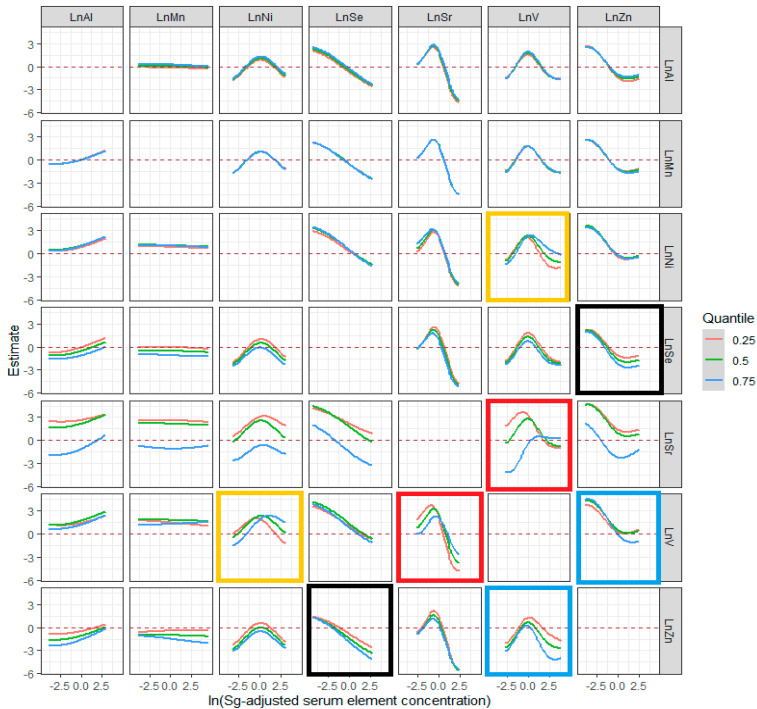
Exposure–response function of a single trace element (column) where the second exposure (row) was fixed at the 25th (red line), 50th (green line) and 75th (blue line) percentile. The model was adjusted for smoking, drinking and family history of EC.

To explore the interaction among the seven trace elements, we further visualized the bivariate exposure–response functions and calculated the interaction effects. An interaction between Sr and V, as well as Ni and V, was also identified for the risk for ESCC, such that the inflection point for Ni increased with elevated levels of V (yellow rectangles), while the inflection point of V was lowered with increasing levels of Sr (red rectangles) (Figure 5). In addition, the potential interactions between V and Zn (blue rectangles) were also observed; when the level of Zn increased, the slope of V became steeper when the other five trace elements were at their median. Moreover, a synergistically protective effect between Se and Zn (black rectangles), two protective factors in the univariate exposure–response analysis, was also found in the analysis.

We finally analyzed the effect of the interaction of these seven trace elements (Figure S2). From the figure, we see that the risk for Sr and V increased by 3.53 and 3.66 units when the other trace elements were fixed at their 25th percentile as compared to when they are fixed at their 75th percentile. As for Se, the risk decreased inversely by 0.963 units.

## 4. Discussion

With the rapid development of the economy and changes in lifestyles, disorders resulting from trace elements have greatly increased, and their effect on cancer prevalence has been constantly studied. In the current study, the impact of multiple serum trace elements in combination on the risk for ESCC, a common pathological type of esophageal cancer, was explored in a case–control study in a southern area of China. Of the 13 trace elements examined, Zn and Se were found to be negatively associated with the incidence of ESCC, and the effect is synergistic. BKMR analysis also indicated the overall effect of the mixture of seven trace metals (Al, V, Mn, Ni, Zn, Se, Sr) and the individual effects of Sr, Se, Zn and V, with strong interactions between V with Ni, Sr and Zn.

Zn has been reported in numerous studies to be a protective factor in papillary thyroid, prostate, breast, lung and other cancers [17,18,19,20,21]. In addition, there is also sufficient evidence supporting a role for Zn deficiency in ESCC, including molecular epidemiological proof [22] and animal models of Zn deficiency in chemical carcinogenesis [23,24], along with the fact that Zn deficiency induces an inflammation gene signature that fuels ESCC development via mR-31, miR-21 and other microRNAs, accompanied by dysregulation of their target genes [25,26,27]. Consistent with the previous studies, low levels of Zn indicate an increased risk for ESCC in a linear dose–response relationship. Interestingly, BKMR analysis showed Se, another element that is associated with a decreased risk for ESCC [28,29], has a synergistic anticancer effect with higher doses of Zn. As a metalloid, Se is an essential trace element in the human body, regulating selenoprotein synthesis, which is involved in antioxidant defense and prevents oxidative stress damage [30,31]. The reports about interactions between Zn and Se in cancer are limited, but a rodent model of Se+Zn supplementation has shown that Zn can suffer from homeostasis mediated by Se, thereby disrupting the metallothionein system and consequent antioxidant function. However, it depended on the concentration of selenium and form of Se. Thus, the result should be verified by epidemiology in the future.

We show Sr is another metal associated with a decreased risk for ESCC. Physiologically, Sr is an important component of bones and teeth, and maintains nerve and muscle membrane excitability [32,33] Higher Sr levels have been shown in cancer patient specimens, such as urinary and serum strontium in breast cancer, as has elevated Sr in the sera of bladder cancer objects [34,35]. However, a case–control study demonstrated the protective effect of Sr in poorly differentiated, malignant breast tumors. Additionally, in lung cancer patients, no significant difference was found in plasma Sr level, and an ecological study did not show different concentrations of serum Sr between high- and low-risk areas of esophageal cancer in Iran [36]. These differences from our study may be explained by the cancer types or exposure doses in different populations, since our study shows an inverted U-shaped dose–response relationship with ESCC in univariate analysis, implying a non-linear association between Sr and ESCC risk, as well as a significant interaction between Sr and V (also weak interactions with Ni or Se, Figure 5). To our knowledge, previous studies have not investigated the mixed effects of Sr in esophageal cancer. Because of extensive environmental pollution and lifestyle changes, humans are undoubtedly exposed to various elements simultaneously, and have antagonistic, additive, synergistic or enhancing effects when taken together, which have been partly proved in previous studies [37,38].

V is a biologically relevant element that incorporates into biomolecules and influences several enzymes [39]. Non-occupational V exposure can come from dietary uptake and air contamination, such as air-suspended particles and smoking [40,41]. Since V is also widely applied in manufacturing, leading to health concerns for occupationally-exposed workers [42], governments have also set exposure limits in vanadium-utilizing/-generating industries. In cancer research, vanadium pentoxide was identified as a possible carcinogen (IARC group: 2b) in 2006, following the demonstration of an increased risk for neoplasms in animals [43]. Subsequent research also indicated that vanadium-pentoxide-induced carcinogenesis was dependent on the mouse strain [44]. In vitro, chronic exposure to V (sodium meta-vanadate) induces malignant phenotypic alterations of immortalized human bronchial epithelial cells [45], and COX-2, an essential enzyme involved in inflammation and constitutively overexpressed in malignancies, can be induced by vanadium pentoxide [46]. However, another epidemiological study including more than four hundred lung cancer patients found no difference between cases and controls. Our biphasic dose–response relationship between serum V level and ESCC risk can explain the above contradictory studies. The efficacy of carcinogens is decided by the ultimate forms, which can be metabolites [47], and different forms of a metal compound can vary in their carcinogenic potential and cellular mechanism [48,49]. Though with high sensitivity and specificity, ICP-MS detected the total concentration of elements but not the specific forms of compounds. Unexpectedly, in the present study, we uncovered significant interactions of V with other elements, i.e., Ni, Zn and Sr, in the bivariate exposure–response analysis (Figure 5). Little of the literature explored the mixed effects between V and other elements in human specimens, which is mainly because of the higher limit of detection of previous research, and the progression of technology and statistical models would help to further uncover the links between them.

Limited epidemiological studies have investigated the association between trace element exposure and risk for ESCC. The only study detected 33 elements in the sera of 100 ESCC patients and identified some elements, e.g., Sr, S, U, P and their combination, in classifying patients and healthy individuals by random forest analysis. Though including fewer elements, our study quantifies the dose–response relationships, as well as the interactions and overall effects of individual pollutants, between element levels and risk for ESCC. Moreover, regional and populational differences may explain some of the variation observed in previous studies. Another study in a southern area of China [50] found higher Cd in ESCC patients, but statistical significance was not found in our study. Specimen differences could be the reason, since Cd has a lower distribution in serum than in whole blood. 

Cigarette smoking, alcohol drinking and a family history of EC are well established as major risk factors for ESCC in Asian populations, and they also act synergistically [51]. In our study, though the related risk or protective effects of some elements (e.g., Al, V, N, Zn, Se and Sr) changed after adjusting the covariates, similar conclusions still held, suggesting a complex etiology for ESCC. Genetic factors (e.g., geography, sex, race, genetic susceptibility and family history) and lifestyle (e.g., tobacco use, alcohol consumption, diet, and hot beverages) concurrently or sequentially influence the development of esophageal carcinoma [52,53]. It is not precisely known how much all these of factors contribute to the malignant transformation of esophageal epithelial cells due to the variety of studied populations and fast changing lifestyles and environments. However, internal exposure levels of trace elements reflect the comprehensive outcomes of multiple factors. Compared with other research, we focused on the complex non-linear relationships and interactions of trace metals in the risk for the development of ESCC. However, inevitably, our study has some important limits. First, causality cannot be fully deduced with an observational study. Furthermore, an additional assessment and examination of pollutants, such as polycyclic aromatic hydrocarbons, should be included due to the complex exposure of environment contaminations. Lastly, since the subjects were recruited in cities of southern China, a high-risk area of ESCC, genetic interactions require consideration and should be pursued in further investigation.

## 5. Conclusions

In summary, using BKMR models, our study finds that serum Se, Sr and V are statistically associated with the risk for ESCC. However, exposure to the combined mixture of these seven trace elements reduces the risk of EC with increasing concentrations. The interaction between Se, Zn V, Sr and Ni, accompanied by different dose–response modes, was also observed. Prospective studies are needed to validate these associations in the future.

## Figures and Tables

**Table 1:** Demographic characteristics of the participants.

Variables	Controls (*n* = 191)	Cases (*n* = 185)	*p*
Age, mean ± SD	62.23 ± 8.86	62.65 ± 7.36	0.908
Sex, *n* (%)			
Male	137 (71.73)	133 (71.89)	0.972
Female	54 (28.27)	52 (28.11)	
Smoking, *n* (%)			
No	126 (65.97)	70 (38.25)	<0.001
Yes	65 (34.03)	113 (61.75)	
Drinking, *n* (%)			
No	144 (75.39)	129 (70.49)	0.200
Yes	47 (24.61)	54 (29.51)	
Family history of EC, *n* (%)			
No	189 (98.95)	163 (89.07)	<0.001
Yes	2 (1.05)	20 (10.93)	

**Table 2:** Serum trace element concentrations (μg/L) in cases and controls.

Elements ^a^	Controls (*n* = 191)	Cases (*n* = 185)	*p*
Al	1097.5 (722.0, 1543.0)	857.2 (461.2, 1576.4)	0.015
V	0.788 (0.566, 1.141)	0.849 (0.673, 1.076)	0.030
Cr	29.1 (20.3, 36.0)	28.9 (19.6, 39.4)	0.833
Mn	14.5 (11.0, 20.0)	11.7 (9.2, 15.5)	<0.001
Co	0.370 (0.278, 0.472)	0.295 (0.219, 0.440)	0.001
Ni	7.7 (3.8, 24.5)	7.9 (4.6, 14.0)	0.953
Cu	1067.5 (973.8, 1203.6)	1094.0 (960.9, 1258.9)	0.239
Zn	1186.3 (1033.5, 1635.4)	904.5 (743.7, 1184.6)	<0.001
As	4.4 (2.0, 7.4)	2.5 (1.4, 5.3)	<0.001
Se	131.5 (116.6, 144.4)	107.4 (96.4, 122.4)	<0.001
Sr	83.1 (61.2, 98.3)	48.8 (41.0, 58.1)	<0.001
Cd	0.384 (0.242, 0.670)	0.331 (0.205, 0.569)	0.026
Pb	105.5 (63.4, 136.7)	105.0 (63.0, 171.3)	0.382

Abbreviations: Al, Aluminum; V, Vanadium; Cr, Chromium; Mn, Manganese; Co, Cobalt; Ni, Nickel; Cu, Copper; Zn, Zinc; As, Arsenic; Se, Selenium; Sr, Strontium; Cd, Cadmium; Pb, Lead. ^a^ The serum trace element concentrations are presented as medians (25th, 75th).

**Table 3:** Odds ratios (ORs) and 95% confidence intervals (95% CIs) for multiple serum trace elements associated with ESCC.

Elements	Variables	Quartile 1	Quartile 2	Quartile 3	Quartile 4	*p* for Trend
Al	Case/control	58/36	49/45	31/63	47/47	
	Model 1	1 (Ref.)	0.71 (0.19, 2.62)	0.26 (0.06, 1.09)	2.73 (0.49, 15.39)	0.419
	Model 2	1 (Ref.)	0.58 (0.14, 2.41)	0.15 (0.03, 0.75)	1.92 (0.30, 12.52)	0.537
V	Case/control	27/67	55/39	61/33	42/52	
	Model 1	1 (Ref.)	3.07 (0.92, 10.32)	6.33 (1.67, 24.02)	0.53 (0.14, 2.00)	0.338
	Model 2	1 (Ref.)	3.72 (0.94, 14.70)	7.16 (1.70, 30.15)	0.50 (0.12, 2.15)	0.243
Mn	Case/control	58/36	51/43	44/50	32/62	
	Model 1	1 (Ref.)	1.39 (0.44, 4.44)	0.73 (0.21, 2.56)	0.21 (0.05, 0.95)	0.075
	Model 2	1 (Ref.)	1.11 (0.31, 3.97)	0.74 (0.19, 2.94)	0.12 (0.03, 0.95)	0.099
Co	Case/control	58/36	48/46	38/56	41/53	
	Model 1	1 (Ref.)	1.16 (0.39, 3.43)	1.27 (0.38, 4.20)	2.55 (0.73, 8.89)	0.939
	Model 2	1 (Ref.)	1.46 (0.45, 4.75)	1.81 (0.46, 7.11)	3.90 (0.93, 16.44)	0.674
Ni	Case/control	54/40	51/43	56/38	38/56	
	Model 1	1 (Ref.)	5.43 (1.74, 16.94)	6.48 (1.95, 21.49)	5.59 (1.30, 23.95)	0.003
	Model 2	1 (Ref.)	5.95 (1.74, 20.33)	4.60 (1.31, 16.22)	3.60 (0.74, 17.48)	0.008
Zn	Case/control	81/13	47/47	23/71	34/60	
	Model 1	1 (Ref.)	0.22 (0.06, 0.75)	0.04 (0.01, 0.17)	0.07 (0.01, 0.41)	0.001
	Model 2	1 (Ref.)	0.39 (0.10, 1.57)	0.04 (0.01, 0.21)	0.12 (0.02, 0.86)	0.004
As	Case/control	63/31	52/42	37/57	15/79	
	Model 1	1 (Ref.)	1.00 (0.33, 3.04)	1.13 (0.35, 3.59)	2.94 (0.78, 11.12)	0.023
	Model 2	1 (Ref.)	0.80 (0.24, 2.63)	0.92 (0.25, 3.32)	2.86 (0.63, 12.96)	0.046
Se	Case/control	81/13	52/42	37/57	15/79	
	Model 1	1 (Ref.)	0.27 (0.08, 0.92)	0.22 (0.06, 0.75)	0.05 (0.01, 0.20)	<0.001
	Model 2	1 (Ref.)	0.18 (0.05, 0.71)	0.19 (0.05, 0.74)	0.06 (0.01, 0.27)	<0.001
Sr	Case/control	75/19	67/27	41/53	2/92	
	Model 1	1 (Ref.)	1.32 (0.44, 3.96)	0.15 (0.05, 0.47)	0.00 (0.00, 0.01)	<0.001
	Model 2	1 (Ref.)	1.11 (0.33, 3.72)	0.11 (0.03, 0.40)	0.00 (0.00, 0.01)	<0.001
Cd	Case/control	56/38	47/47	39/55	43/51	
	Model 1	1 (Ref.)	0.46 (0.12, 1.73)	1.24 (0.29, 5.29)	1.49 (0.34, 6.45)	0.381
	Model 2	1 (Ref.)	0.43 (0.10, 1.79)	1.12 (0.23, 5.53)	1.66 (0.35, 7.80)	0.308

Note: Model 1 is unadjusted. Model 2 is adjusted for smoking, drinking and family history of EC.

## Data Availability

The data presented in this study are available in this article and supplementary material.

## Supplementary Materials

The following supporting information can be downloaded at: https://www.mdpi.com/article/10.3390/cancers14174239/s1, Figure S1: Posterior inclusion probability (PIP) for the included trace elements; Figure S2: Interaction effects of the 7 trace elements; Table S1: Clinical information of the ESCC patients (N = 185); Table S2: Collinearity diagnostics of the 13 trace elements; Table S3: Odds ratios (ORs) and 95% confidence intervals (95% CIs) for individual serum trace elements associated with ESCC.
