# Supplementary material for: Association between Serum Level of Multiple Trace Elements and Esophageal Squamous Cell Carcinoma Risk: A Case–Control Study in China
Source: Cancers (Basel). 2022 Aug 31;14(17):4239. doi: 10.3390/cancers14174239 (PMC9455051; doi:10.3390/cancers14174239)
Supplement: Supplementary file 1 [file cancers-14-04239-s001.zip › cancers-1838382-supplementary.pdf]

Supplementary Materials:

Table S1. Clinical information of the ESCC patients (N=185)

Variables	*Cases, n (%)*
Location, n=184	
Upper	*33(17.9)*
Middle	*111(60.4)*
Lower	*40(21.7)*
pGrade, n=183	
G1	*45(24.6)*
G2	*61(33.3)*
G3	*72(39.3)*
X	*5(2.8)*
pT, n=183	
Tis/T1	*19(10.4)*
T2	*24(13.1)*
T3	*56(30.6)*
T4	*84(45.9)*
pN, n=183	
N0	*79(43.2)*
N1	*62(33.9)*
N2	*31(16.9)*
N3	*11(6.0)*
pTNM, n=183	
0	*1(0.6)*
I	*17(9.3)*
II	*37(20.2)*
III	*84(45.9)*
IV	*44(24.0)*

Table S2. Collinearity diagnostics of the 13 trace elements

Element	Tolerance	VIF
LnAl	0.322	3.106
LnV	0.649	1.542
LnCr	0.400	2.503
LnMn	0.499	2.005
LnCo	0.767	1.304

LnNi	0.559	1.79
LnCu	0.928	1.077
LnZn	0.487	2.055
LnAs	0.779	1.284
LnSe	0.701	1.426
LnSr	0.56	1.787
LnCd	0.449	2.227
LnPb	0.266	3.760

Abbreviations: VIF, variance inflation factor.

Table S3. Odds ratios (ORs) and 95% confidence intervals (95% CIs) for individual serum trace elements associated with ESCC

Elements	Variables	Quartile 1	Quartile 2	Quartile 3	Quartile 4	*P* for trend
	Case/control	58/36	49/45	31/63	47/47	
Al	Model 1	1 (Ref.)	0.68(0.38,1.21)	0.31(0.17,0.56)	0.62(0.36,1.11)	0.019
	Model 2	1 (Ref.)	0.67(0.36,1.25)	0.29(0.15,0.55)	0.62(0.33,1.15)	0.026
	Case/control	27/67	55/39	61/33	42/52	
V	Model 1	1 (Ref.)	3.50 (1.91,6.42)	4.59(2.48,8.55)	2.00(1.10,3.67)	0.019
	Model 2	1 (Ref.)	3.81(1.97,7.36)	5.06(2.60,9.83)	2.04 (1.06,3.94)	0.027
	Case/control	48/46	46/48	39/55	52/42	
Cr	Model 1	1 (Ref.)	0.92(0.52,1.63)	0.68(0.38,1.21)	1.18(0.67,2.11)	0.818
	Model 2	1 (Ref.)	1.13(0.61,2.09)	0.83(0.45,1.54)	1.28(0.69,2.38)	0.675
	Case/control	58/36	51/43	44/50	32/62	
Mn	Model 1	1 (Ref.)	0.74(0.41,1.32)	0.55(0.31,0.98)	0.32(0.18,0.58)	<0.001
	Model 2	1 (Ref.)	0.70(0.38,1.31)	0.59(0.32,1.09)	0.29(0.15,0.56)	<0.001
	Case/control	58/36	48/46	38/56	41/53	
Co	Model 1	1 (Ref.)	0.65(0.36,1.16)	0.42(0.24,0.76)	0.48(0.27,0.86)	0.005
	Model 2	1 (Ref.)	0.69(0.37,1.26)	0.43(0.23,0.82)	0.53(0.28,0.97)	0.018
	Case/control	40/54	51/43	56/38	38/56	
Ni	Model 1	1 (Ref.)	1.60(0.90,2.85)	1.99(1.11,3.56)	0.92(0.51,1.64)	0.963
	Model 2	1 (Ref.)	1.71(0.93,3.17)	2.04(1.10,3.78)	0.86(0.46,1.61)	0.834
	Case/control	48/46	39/55	49/45	49/45	
Cu	Model 1	1 (Ref.)	0.68(0.38,1.21)	1.04(0.591.85)	1.04(059,1.85)	0.549
	Model 2	1 (Ref.)	0.65(0.35,1.21)	1.31(0.70,2.43)	1.27(0.69,2.35)	0.159
	Case/control	81/13	47/47	23/71	34/60	
Zn	Model 1	1 (Ref.)	0.16(0.08,0.33)	0.05(0.03,0.11)	0.09(0.04,0.19)	<0.001
	Model 2	1 (Ref.)	0.19(0.09,0.40)	0.05(0.02,0.11)	0.10(0.05,0.21)	<0.001
	Case/control	63/31	52/42	37/57	15/79	
As	Model 1	1 (Ref.)	0.61(0.34,1.10)	0.28(0.15,0.51)	0.31(0.17,0.56)	<0.001
	Model 2	1 (Ref.)	0.57(0.30,1.07)	0.25(0.13,0.47)	0.29(0.15,0.55)	<0.001
	Case/control	81/13	52/42	37/57	15/79	
Se	Model 1	1 (Ref.)	0.20(0.10,0.41)	0.10(0.05,0.21)	0.03(0.01,0.07)	<0.001
	Model 2	1 (Ref.)	0.18(0.09,0.38)	0.11(0.05,0.23)	0.04(0.02,0.08)	<0.001
	Case/control	75/19	67/27	41/53	2/92	
Sr	Model 1	1 (Ref.)	0.63(0.32,1.23)	0.20(0.10,0.38)	0.01(0.00,0.02)	<0.001
	Model 2	1 (Ref.)	0.63(0.31,1.29)	0.19(0.10,0.38)	0.01(0.00,0.02)	<0.001
	Case/control	56/38	47/47	39/55	43/51	
Cd	Model 1	1 (Ref.)	0.68(0.38,1.21)	0.48(0.27,0.86)	0.57(0.32,1.02)	0.031
	Model 2	1 (Ref.)	0.80(0.43,1.48)	0.48(0.26,0.90)	0.57(0.31,1.07)	0.030
	Case/control	47/47	46/48	36/58	56/38	
Pb	Model 1	1 (Ref.)	0.96(0.54,1.70)	0.62(0.35,1.11)	1.47(0.83,2.63)	0.433
	Model 2	1 (Ref.)	0.91(0.49,1.69)	0.63(0.34,1.17)	1.60(0.86,2.97)	0.296

Note: Model 1 is unadjusted. Model 2 is adjusted for smoking, drinking and family history of EC.

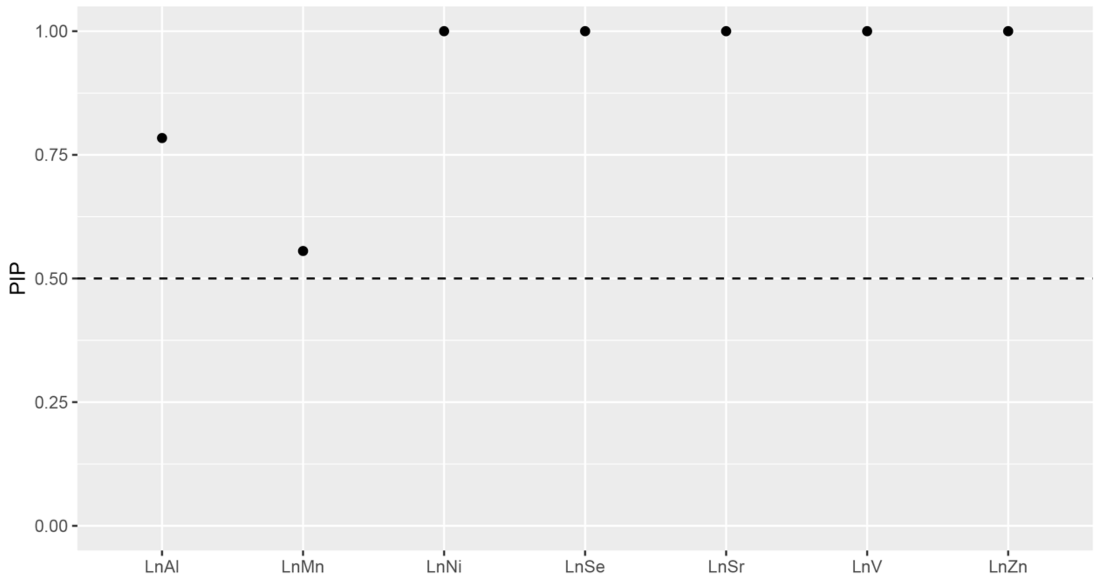

Figure S1. Posterior inclusion probability (PIP) for the included trace elements. Abbreviations: Al, Aluminum; V, Vanadium; Mn, Manganese; Co, Cobalt; Ni, Nickel; Zn, Zinc; As, Arsenic; Se, Selenium; Sr, Strontium; Cd, Cadmium. The PIPs can be thought of as a measure of the variables' importance, with higher values (closer to 1) indicating higher importance, and lower values (closer to 0) indicating lower importance.

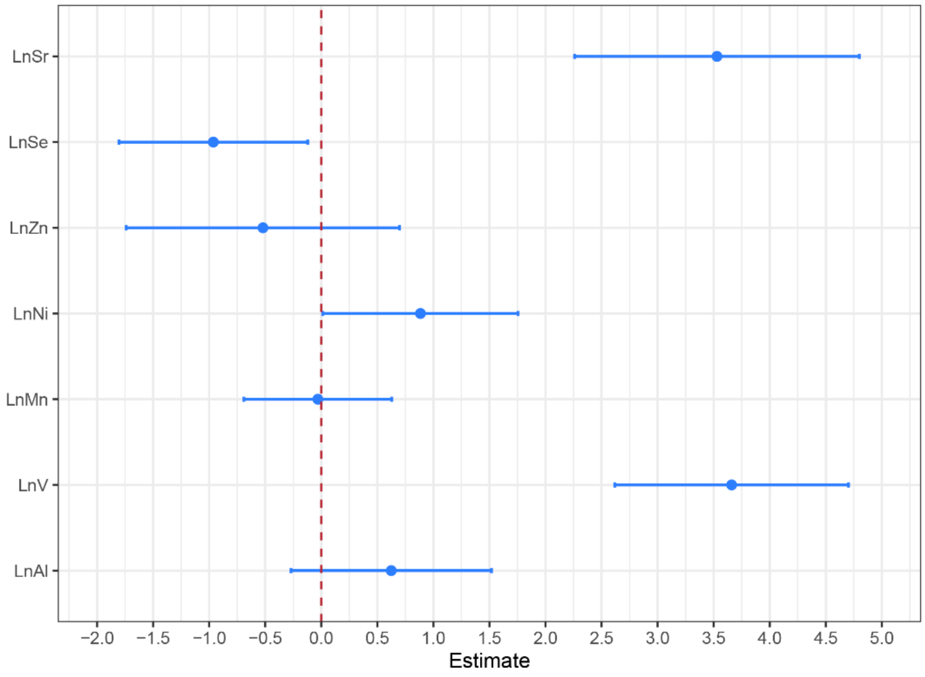

Figure S2. Interaction effects of the seven trace elements. Each individual effect was compared when the concentrations of other trace elements were fixed to their 75th percentile to when their concentrations were fixed to their 25th percentile.
